# A Proposed Methodology to Control Body Temperature in Patients at Risk of Hypothermia by means of Active Rewarming Systems

**DOI:** 10.1155/2014/136407

**Published:** 2014-11-17

**Authors:** Silvia Costanzo, Alessia Cusumano, Carlo Giaconia, Sante Mazzacane

**Affiliations:** ^1^DEIM, Palermo University, Viale delle Scienze Ed. 9, 90128 Palermo, Italy; ^2^CIAS Laboratory, Research Centre on Physical, Chemical and Microbiological Pollution in High Sterile Rooms, Department of Architecture, Ferrara University, Via Quartieri 8, 44121 Ferrara, Italy

## Abstract

Hypothermia is a common complication in patients undergoing surgery under general anesthesia. It has been noted that, during the first hour of surgery, the patient's internal temperature (*T*_core_) decreases by 0.5–1.5°C due to the vasodilatory effect of anesthetic gases, which affect the body's thermoregulatory system by inhibiting vasoconstriction. Thus a continuous check on patient temperature must be carried out. The currently most used methods to avoid hypothermia are based on passive systems (such as blankets reducing body heat loss) and on active ones (thermal blankets, electric or hot-water mattresses, forced hot air, warming lamps, etc.). Within a broader research upon the environmental conditions, pollution, heat stress, and hypothermia risk in operating theatres, the authors set up an experimental investigation by using a warming blanket chosen from several types on sale. Their aim was to identify times and ways the human body reacts to the heat flowing from the blanket and the blanket's effect on the average temperature *T*_skin_ and, as a consequence, on *T*_core_ temperature of the patient. The here proposed methodology could allow surgeons to fix in advance the thermal power to supply through a warming blanket for reaching, in a prescribed time, the desired body temperature starting from a given state of hypothermia.

## 1. Introduction

Hypothermia in patients subject to surgery under total or local anesthesia is linked to the inhibition of their thermoregulatory system and to their exposure to the operating room environment. Several studies [1–3] have shown how a patient, undergoing more than one hour of surgery, reaches hypothermia conditions under the combined effect of anesthetizing drugs, possible exposure to cold air draughts, infusion of liquids (including blood) at normal room temperature, and increased heat losses to the outside environment due to the surgical incision or even internal organs exposed to air. Hypothermia can lead to various complications such as shivering, longer postoperative wakeup times, compromised coagulation, ischemic heart events, and lowered immune defenses against surgical wound infections [4–7].

Hypothermic states can be continuously monitored through temperature *T*_body_ given as the weighted average of internal body temperature and of mean whole body *T*_skin_, in accordance with Brock and Zeisberger's equation [8]:

(1) $$\begin{array}{c} T_{\text{body}}=0.87+0.13T_{\text{skin}}. \end{array}$$

To assess *T*_core_, the currently used method uses a probe inserted down to the esophagus. An esophageal probe is indeed highly invasive, so anesthetists, through their personal experience, prefer to adopt this technique only in very complex surgery, avoiding it for simple, routinary procedures. Given the difficulty of measuring core temperature at esophageal level, correlations between core temperature and those of other body areas have been sought: it was noted, in particular, that between *T*_core_ measured in the esophagus and temperature measured on the tympanic membrane (*T*_tympanic_) variance is only −0.1°C [9–12].

**Figure 1:**
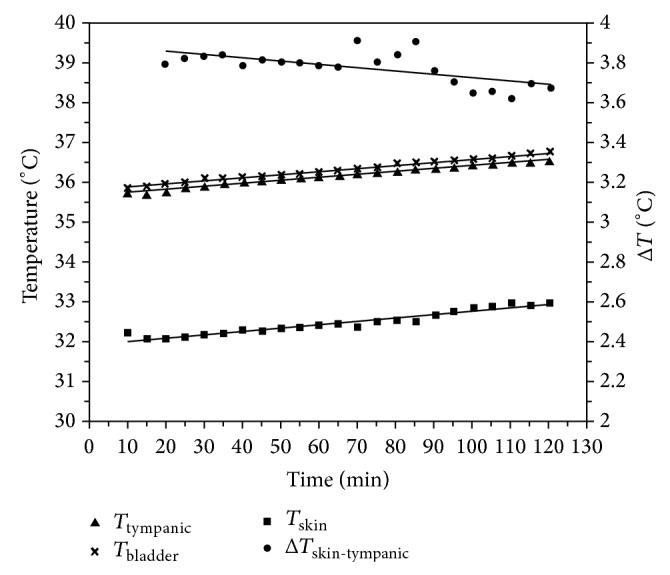
Courses of *T*_skin_, *T*_tympanic_, *T*_bladder_, and average differences to *T*_skin_ according to Tran and others' study.

For *T*_skin_ calculation used in (1), the literature suggests many formulae: the most trustworthy seem to be those of Hardy and Dubois [13] with 7 measurement points, of Houdas and Ring [14] with 5 points, and of Olesen with 3 points [15, 16]. Trans and others' [17] recent paper on a group of individuals of different age and sex gives a correlation between mean *T*_skin_ and the tympanic and blood temperature at urinary bladder level. The graph in Figure 1 refers to different temperature courses and the mean differences between them.

**Figure 2:**
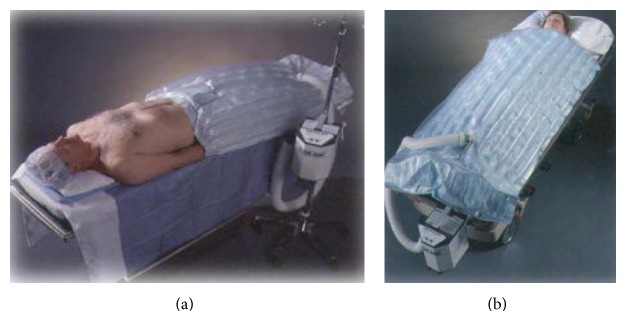
Warming blanket used for localized rewarming.

**Figure 3:**
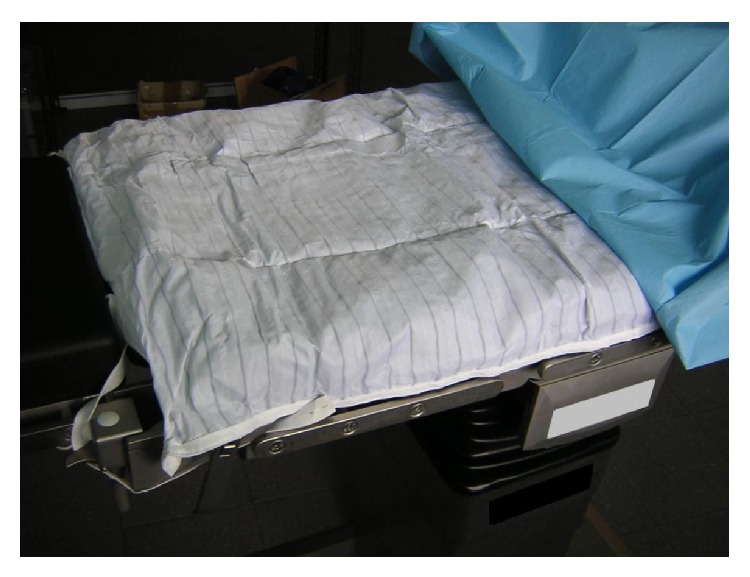
Warming blanket.

Whether preventing or recovering from hypothermia, either passive type systems (like blankets holding back body heat spread) or active ones (thermal blankets as in Figures 2(a), 2(b) and 3 electric or hot-water mattresses, forced hot air, warming lamps, etc.) are used.

This paper reports the data of the authors, who aimed to identify times and ways the human body reacts to heat flowing out from a warming blanket. Tran et al.'s [17] correlations were used to work out a methodology to determine best timings to switch on the warming blanket, in order to control patient hypothermia safely. The method allows surgeons to predict how much heat must be supplied through the warming blanket, in order to recover, in a prescribed time, the desired body temperature, starting from a given state of hypothermia.

## 2. Materials and Methods

In this study a warming blanket, commonly used in hospitals, was used and it was subjected to experimental tests in the laboratories of Palermo University. The warming blanket, made of polyester fiber including carbon microfilaments, develops the required heat flux by using a low voltage supply. An electronic power supply allows adjusting the thermal flux on 3 levels.

Two series of lab tests were performed during the study: measurements upon the warming blanket and measurements on human body.

1. The first series of tests aimed to assess the uniformity of temperature throughout blanket surface and to evaluate the thermal power at each of the 3 different set points of the electronic power supply. In this connection, surface temperatures and heat flows were measured in suitably chosen points of the blanket.
2. The second series of tests, carried on subjects lying on the blanket, aimed to find out the average temperature of skin (*T*_skin_) and, after that, the subject's *T*_core_ and *T*_body_, with the aim assessing the power supply switch-on intervals needed to recover the patient back to normal thermal conditions. Therefore, surface temperatures were measured on some of the subject's body points suitably chosen.

During the measurements, the air temperature and the relative humidity of the ambient where the test was carried out were measured as well.

### 2.1. Measurement Equipment

**Figure 4:**
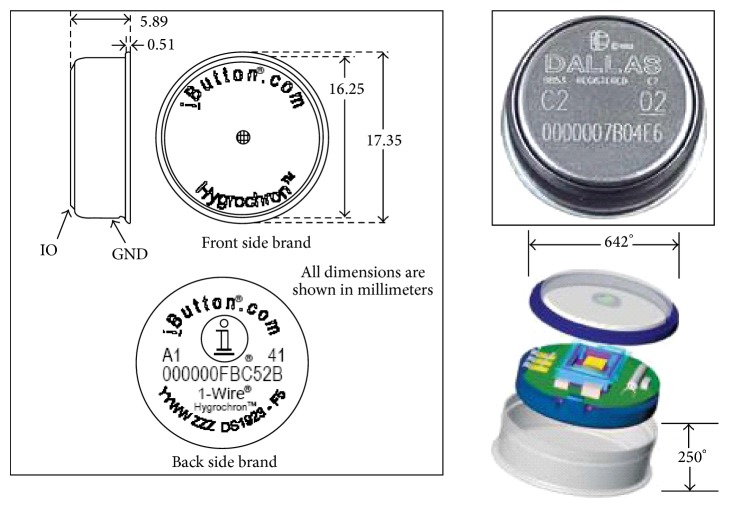
Description and photographs of i-Button sensor.

The environmental data and the surface temperatures were collected by using two types of i-Button microrecorders (made by Dallas Semiconductors) (see Figure 4): DS1921H model (High Resolution Thermochron, Human Temperature Range) to measure surface body temperature and DS1923 model (Humidity and Temperature Logger) [19].

For measurement of thermal flows, some thermopiles (model PU 22T of the TNO of Delft) were used.  Table 1 shows their characteristics.

### 2.2. Measurements on the Warming Blanket

**Figure 5:**
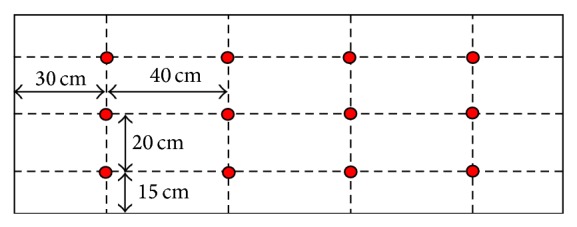
Placement of i-Button microrecorders on warming blanket.

To measure surface temperatures, twelve i-Button sensors (model DS1921H) were placed on the warming blanket (see Figure 5). Tests were made by placing the blanket between two layers of insulating material with well-known conductive properties.

After waiting for the thermal steady state, the electronic supply was set subsequently on the three set points: each time waiting for the stationary state to be reached. As noted above, the values of environmental temperature and relative humidity, along with thermal flow, were recorded. In this way, heat fluxes (in correspondence with every set point value) flowing from the warming blanket were assessed. In Table 2 are reported mean values of standard deviation relative to temperature and relative humidity of outside air and measured by the twelve microrecorders in steady state along with the corresponding values of thermal flux.

The standard deviation *s* relative to the whole data population was worked out according to the following formula:

(2) $$\begin{array}{c} s=\sqrt{\frac{\sum_{i=1}^{N}{\left(x_{i}-\overset{-}{x}\right)}^{2}}{N-1}}, \end{array}$$

where *x*_*i*_ is the generic sample of temperature, $\overset{-}{x}$ is the mean value of temperature samples, and *N* is the total number of samples. The data show a good uniformity of the surface temperature all over the warming blanket. It is noteworthy that the standard deviation value, though rising from set point 1 to set point 3, keeps within absolutely tolerable limits.

### 2.3. Measurements on Human Body

The second set of tests aimed to verify the warming effect produced by the blanket in maintaining and restoring the normal temperature in a patient. Being unable to take measurements in a real operating room, efforts were made to evaluate temperature gradients on various body parts of some patients lying supine on the warming blanket, in order to test the time course of average skin temperature *T*_skin_ relative to different set points of the power supply. Tran's correlations helped us to obtain the corresponding courses of *T*_core_ and then of *T*_body_. The temperature and humidity of the room, with doors and windows closed, were measured with DS1923 i-Button sensors.

**Figure 6:**
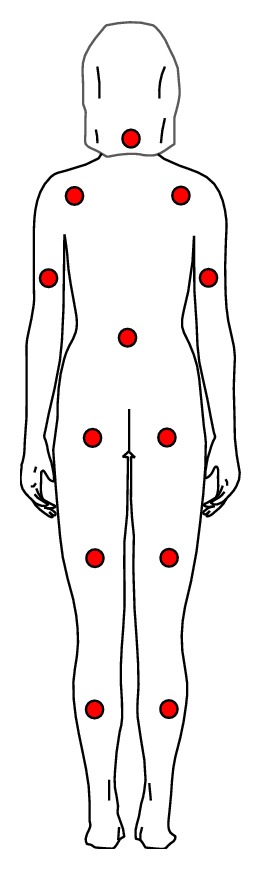
Placement of i-Button sensors on the subject's back.

Figure 6 shows where the i-Button microrecorders (model DS1921H) were positioned on the human body, stuck with heat-conducting jelly, and fixed with perspiration-proof surgical adhesive tape. Two other sensors were applied to chest and forearm, along with one on the calf, to measure *T*_skin_ at the 3 measuring points suggested by Olesen [16], a formula the authors already used and tested in a previous paper on heat stress experienced by a surgical team inside an operating room [20, 21].

Four measurement tests were made for each individual, one for each set point of the warming blanket's power supply. The tests were carried out under the following conditions:

1. warming blanket switched OFF: initial values of surface temperatures on the points marked in Figure 6;
2. warming blanket switched ON (set points 1, 2, and 3): time course of surface temperatures until a stationary state is reached, with thermostat set at each available set point.

**Figure 7:**
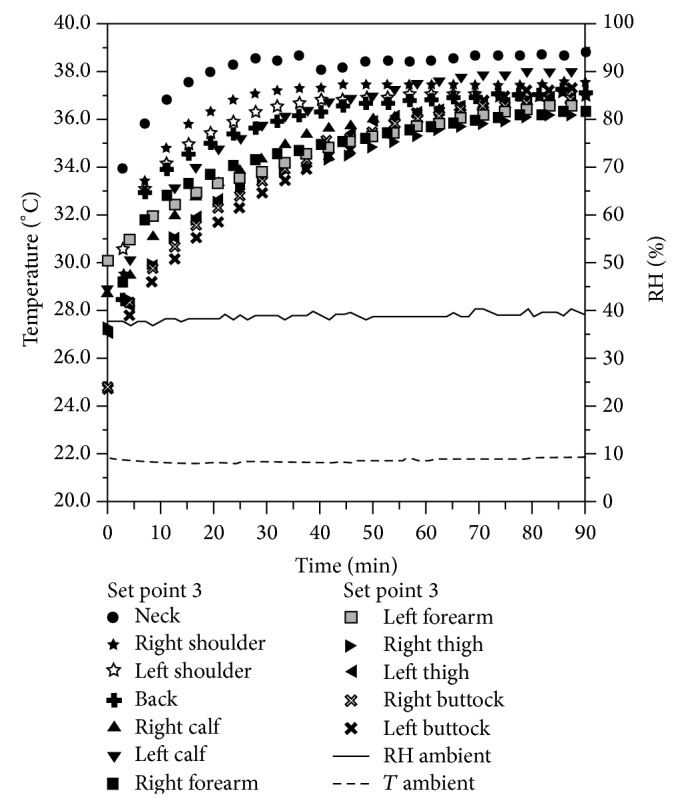
Courses of surface temperatures in different body parts at set point 3.

This paper reports, as an example, the data referring to a female individual weighing about 55 kilograms, approximate height 1.60 m.  Figure 7 shows a measurement taken using a warming power of 71.41 Watt (set point 3).

On the graph can be observed the temperature time behavior in correspondence with different body parts. Temperature and humidity in the test environment are also reported.

**Figure 8:**
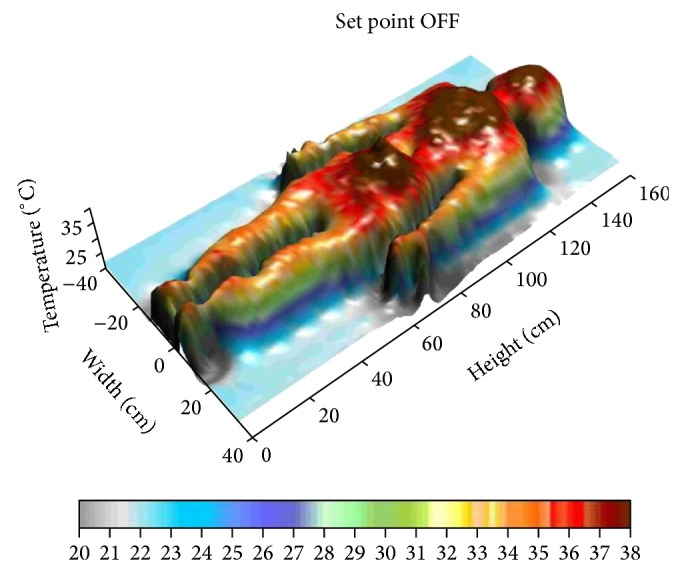
Set point OFF.

**Figure 9:**
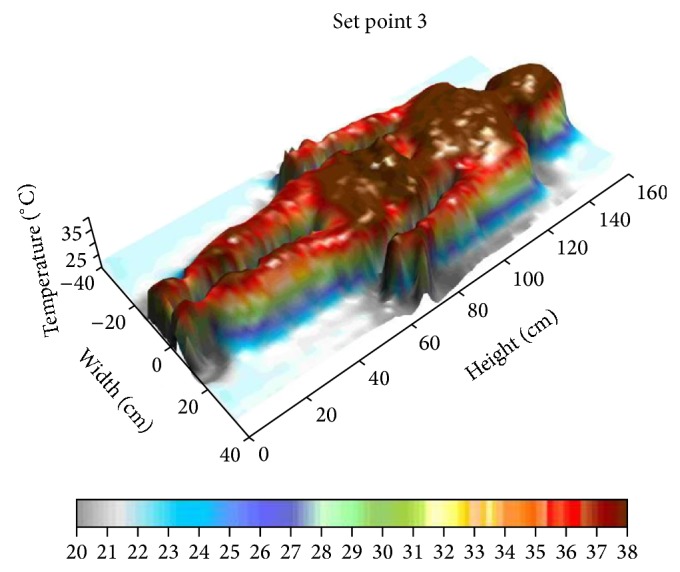
Set point 3.

The three dimensional graphs (Figures 8 and 9) show how the person taken as example gets warmer in her central part as the power supplied by the warming blanket goes up.

## 3. Results and Discussion

To set up a methodology for assessing the power levels that the warming blanket has to supply in order to restore the patient to normal conditions, the experimental data were elaborated with the aim of determining the thermal time constant for each different body part. To assess the temperature rise times in the diverse body parts, an interpolation was worked out of the values of surface temperatures measured in the human body using an equation similar to that used for heat transmission in systems with concentrated parameters:

(3) $$\begin{array}{c} T\left(\tau \right)=C+Ae^{\left(-B\tau \right)}. \end{array}$$

In formula (3) the term *A* is linked to initial temperature value, 1/*B* represents the time constant, and *C* is the norm of temperature value. All the terms refer to a single body point. The time constant value at a particular body point depends on total heat resistance and on the body tissues' heat capacity. In particular,

(4) $$\begin{array}{c} B=\frac{1}{RC}\left[{\text{s}}^{-1}\right], \end{array}$$

where *R* is thermal resistance calculated for a single body part [K/W] and *C* is thermal capacity for a single body part [J/K].

**Figure 10:**
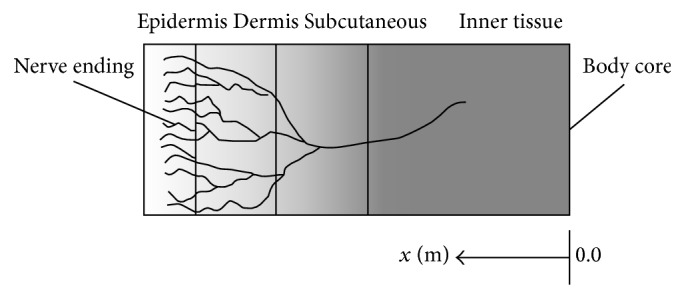
Schematic geometry of three-layer skin structure.

Total thermal resistance is obtained from the sum of contact resistance of blanket with resistances of epidermis and layers that make up the human skin. In this regard, Lv and Liu [18] suggested an electric model based on four tissue layers (see Figure 10).  Table 3 shows the values the authors presume for the skin's geometry and thermophysical nature.

**Figure 11:**
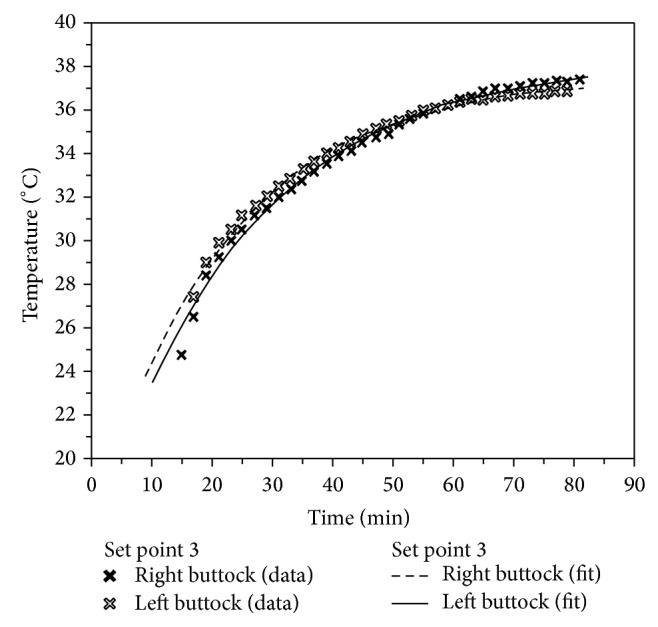
Interpolation function *T*(*τ*) on subject's right and left hip.

As it is known the time constant value helps to define the time for reaching the steady state conditions.  Figure 11 shows the course of interpolation function *T*(*τ*) on right and left sides of subject's hip.

Table 4 shows inverse values of the time constant for the different body parts and for the different set points obtained by interpolations made according to (3).

**Figure 12:**
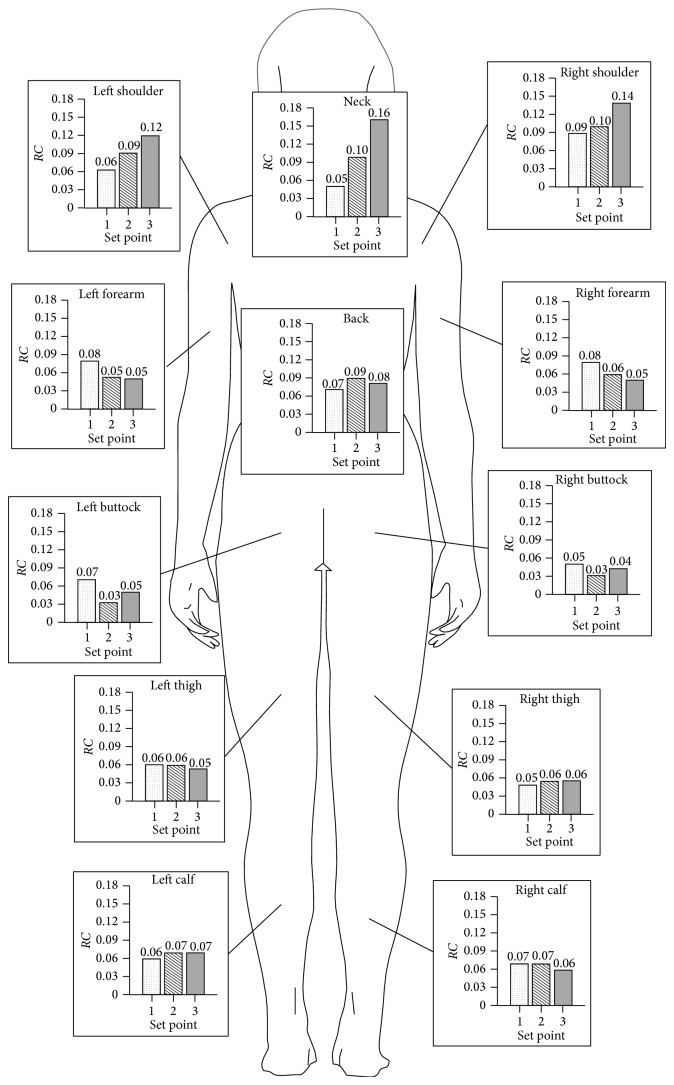
Values of *B* [s^−1^] for the different body parts and for the warming blanket's different set points.

Figure 12 gives the distribution of *B* values in the subject's body.

**Figure 13:**
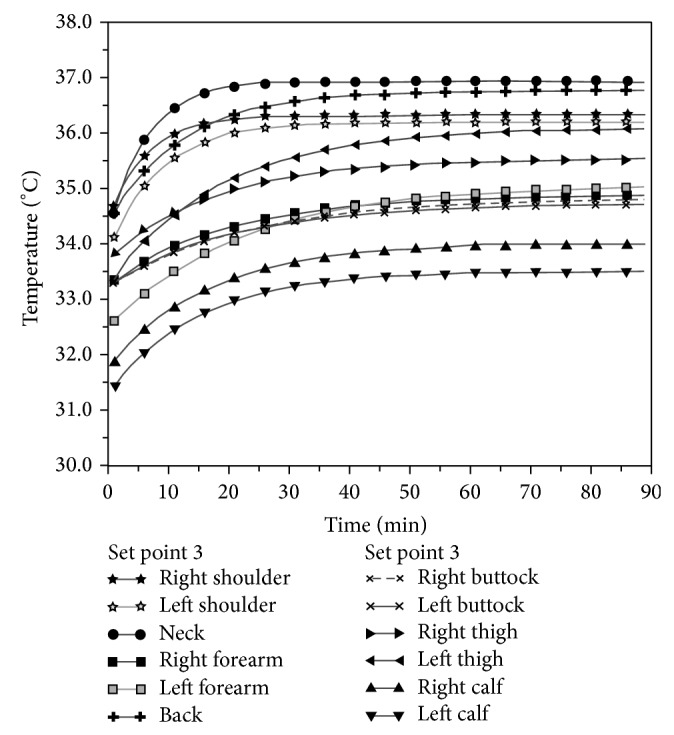
Interpolated course of the temperatures shown in Figure 7.

Time constants relating to various body parts make it possible, through (3), to get temperature courses in the different body parts for any kind of final value, starting from any initial value.  Figure 13 shows the temperature courses of different body parts of Figure 7, calculated through interpolation equations that use time constants reported in Table 4 and the experimental values of initial and final temperatures.

The interpolation equations for different body parts make it possible to test the time course of temperature *T*_skin_, calculated through Olesen's formula with 3 measurement points [16]:

(5) $$\begin{array}{c} T_{\text{skin}}=\text{0,5}a+\text{0,14}b+\text{0,36}c, \end{array}$$

where *a* is chest temperature, *b* is forearm temperature, and *c* is calf temperature.

Comparisons made among Tran and others' study lead to an evaluation of *T*_core_:

(6) $$\begin{array}{c} T_{\text{core}}=\left(-\text{0,00164}\tau +\text{3,9}\right)+T_{\text{skin}}. \end{array}$$

**Figure 14:**
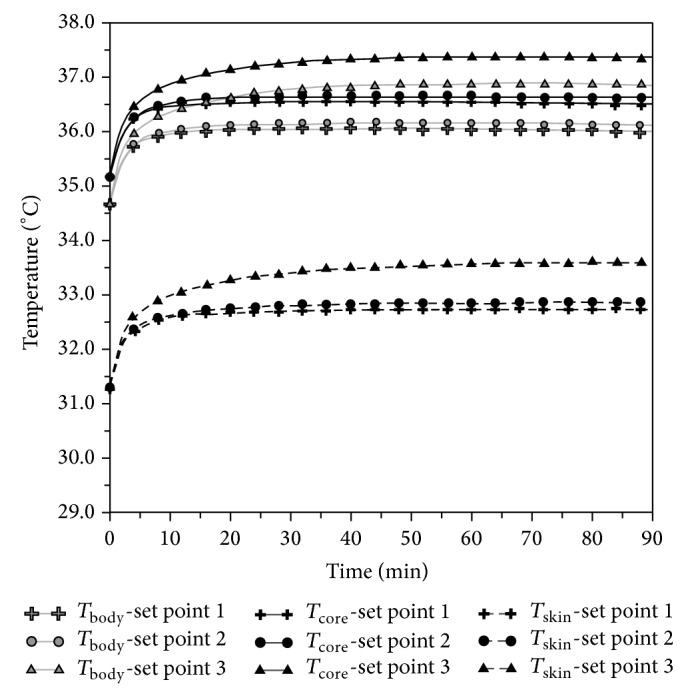
*T*_skin_, *T*_core_, and *T*_body_ for the three set points.

Finally, from (1), the *T*_body_ used as index of hypothermia state can be worked out.  Figure 14 shows, for each set point, the time courses of *T*_skin_, *T*_core_, and *T*_body_.

With the aim of assessing a relationship valid for the whole body, the global value of *B* = 1/*RC* has been obtained. The *RC* term stands for the time constant of the whole body acquired by interpolation of the course of *T*_body_ calculated by means of temperatures measured in various body parts. In the example, the average value of *B* for the subject tested came out as equal to 0.27 [s^−1^].

So, following these hypotheses, the equation of temperature course for the whole body as function of time can be written in the following way:

(7) $$\begin{array}{c} T_{\text{body}}=T_{\text{final}\;\text{body}}+\left(T_{\text{initial}\;\text{body}}-T_{\text{final}\;\text{body}}\right)e^{-\left(\tau /RC\right)}. \end{array}$$

Equation (7), for a given *RC* value, can help us to work out times for reaching temperature *T*_final  body_ value, starting from a given *T*_initial  body_ value. From the test values of heat power delivered by the power supply at each set point and of initial and final *T*_body_ values, the following equation is obtained:

(8) W=−0.256+12,385Tfinal  body−Tinitial  body +8,393Tfinal  body−Tinitial  body2.

**Figure 15:**
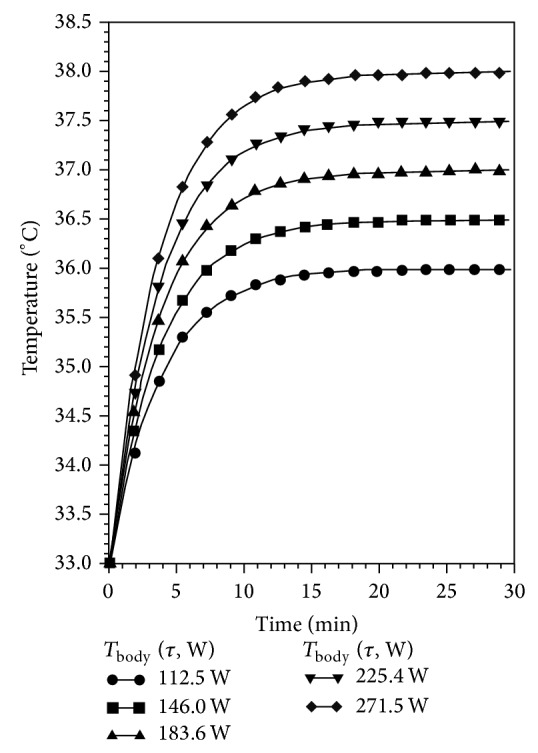
Example of assessing *T*_final  body_ as a function of time (*T*_initial  body_) and of power *W*.

Equation (8) helps us to decide the power to be supplied to the warming blanket, so as to recover the patient in a fixed time from a given state of hypothermia. From (7) and (8) can be worked out a graph to determine electrical power *W* that the warming blanket has to supply for a prescribed time, to reach the desired body temperature *T*_final  body_, starting from a given state of hypothermia *T*_initial  body_. Taken as an example, with data referring to the subject under test, the graph in Figure 15 can be drawn, assuming 33°C as initial body temperature.

## 4. Conclusions

In this paper an experimental method is described, in order to assess the recovery times for patients who undergo surgery, using an active warming system. This study is a part of a wider research carried out by the authors into heat stress states in highly sterile conditions.

The proposed methodology (developed through experimental trials carried out in the laboratories of the DEIM of Palermo University) made use of a warming blanket, with a heat regulator, commonly used in hospital facilities and has been developed with the following steps.

- Measure the time course of surface temperature in different parts of a human body, using high sensitivity microrecorders.
- Calculate localized values of specific time constants at each of the chosen body points, with reference to the heat flux provided by the warming blanket, interpolating the experimental data.
- Assess *T*_body_ temperature with the aid of relationships found in the literature.
- Assess the time constant of the subject's whole body.
- Assess the relationship between *T*_final  body_ and *T*_initial  body_ and time constant *τ*.
- Assess the relationship between power *W* provided by the warming blanket and the difference (*T*_final  body_ and *T*_initial  body_).
- Assess the relationship among power *W*, the difference (*T*_final  body_ and *T*_initial  body_), and time constant *τ*.

The methodology described above allows us to assess the heat flux to apply with the warming blanket, to reach, within a prescribed time, the desired body temperature *T*_final  body_, starting from a given state of hypothermia *T*_initial  body_. If one applies this procedure to a large number of patients (with different ages, sex, and body weight), one will be able to devise a general purpose relationship, as a useful tool for improving patient safety during surgical operations.

Nevertheless, we still need to ascertain whether using active means of rewarming inside an operating theatre contributes to changing the sterile state of the room. The authors, in their next study, propose to investigate the effects of rewarming means in the environment close to the operating theatre and, above all, with regard to how air movements get generated and bring pathogens that could infect the surgical wound.

## Figures and Tables

**Table 1:** Thermopiles model PU 22T of the TNO (Delft).

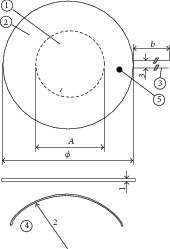					
Dimension of PU-T category of 1 mm thickness: sensitive area (1), guard (2), fixed wires (3), minimum bending radius (4),					
and optional temperature sensor (5)					
Note: PU-T sensors can be bent but are quite rigid. Installation typically requires taping.					

Model		PU 11T	PU 22T	PU 32T	PU 43T

Thickness	mm	1	1	1	1
Overall diameter	mm	25	50	75	100
Dimensions sensitive area	mm^2^	*⊘* 15	*⊘* 30	*⊘* 30	*⊘* 55
Sensitivity (nominal)	*μ*V/mm^−2^	8	30	30	150
Electrical resistance	Ohm	400	1700	1700	7000
Filling material		PU	PU	PU	PU
Temperature range	°C	−20 to +90	−20 to +90	−20 to +90	−20 to +90
Thermal resistance	m^2^K/W	0.004	0.004	0.004	0.004
Expected accuracy	%	+5/−5% @ 20°C, temperature dependence typically 0.17%/K			
Cable connection		Fixed wires 2 m			
Minimum bending radius	mm	15	25	40	50

**Table 2:** Environmental parameters, average surface temperature, standard deviation of temperature measured by microrecorders in stationary state, and power values.

Set point	OFF	1	2	3
External temperature [°C]	26.8	27.8	27.8	27.9
RH [%]	48.2	47	48.2	48.6
Mean surface temperature [°C]	26.4	28.2	29.4	32.0
Standard deviation *s*	0.160	0.246	0.389	0.924
Power [W]	0	25.55	41.93	71.41

**Table 3:** Thermophysical properties of the skin in accordance with Lv and Liu [18].

	Specific heat *C* (J/kg K)	Thermal conductivity *K* (W/m K)	Thickness *L* (m)	Density *ρ* (kg/m^3^)
Epidermis	3578–3600	0.24	80 × 10^−6^	1200
Dermis	3200–3400	0.45	0.002	1200
Subcutaneous	2288–3060	0.19	0.01	1000
Inner tissue	4000	0.5	—	1000

**Table 4:** Values of B [s^−1^] for the different body parts.

Set point	1	2	3
Neck	0.05	0.1	0.16
Shoulder Dx	0.09	0.1	0.14
Shoulder Sx	0.063	0.09	0.12
Forearm Dx	0.08	0.06	0.05
Forearm Sx	0.08	0.052	0.05
Back	0.07	0.09	0.081
Pelvis Dx	0.05	0.031	0.043
Pelvis Sx	0.07	0.032	0.05
Thigh Dx	0.05	0.056	0.056
Thigh Sx	0.06	0.06	0.054
Calf Dx	0.07	0.07	0.061
Calf Sx	0.06	0.07	0.07
